# The absence of muscle segment homeobox 2 leads to the pyroptosis of ameloblasts by inducing squamous epithelial hyperplasia in the enamel organ

**DOI:** 10.1111/jcmm.16646

**Published:** 2021-05-26

**Authors:** Juanjuan Zhang, Ying Xu, Ying Zhao, Jingkun Bai, Mengge Xu, Chuanji Li, Jinyue Li, Yong Ren, Chang Xu, Yuguang Gao, Yan Sun, Xiaoying Liu

**Affiliations:** ^1^ Department of Oral Biology School of Bioscience and Technology Weifang Medical University Weifang China; ^2^ Department of Pediatric Dentistry Binzhou Medical University Yantai China

**Keywords:** ameloblast, enamel development, MSX2, pyroptosis, squamous epithelial hyperplasia

## Abstract

Muscle segment homeobox 2 (MSX2) has been confirmed to be involved in the regulation of early tooth development. However, the role of MSX2 has not been fully elucidated in enamel development. To research the functions of MSX2 in enamel formation, we used a *Msx2* ^−/−^ (KO) mouse model with no full *Msx2* gene. In the present study, the dental appearance and enamel microstructure were detected by scanning electron microscopy and micro‐computed tomography. The results showed that the absence of *Msx2* resulted in enamel defects, leading to severe tooth wear in KO mice. To further investigate the mechanism behind the phenotype, we performed detailed histological analyses of the enamel organ in KO mice. We discovered that ameloblasts without *Msx2* could secrete a small amount of enamel matrix protein in the early stage. However, the enamel epithelium occurred squamous epithelial hyperplasia and partial keratinization in the enamel organ during subsequent developmental stages. Ameloblasts depolarized and underwent pyroptosis. Overall, during the development of enamel, MSX2 affects the formation of enamel by regulating the function of epithelial cells in the enamel organ.

## 1 INTRODUCTION

Enamel is the tissue with the highest degree of mineralization in the human body. Its developmental process includes the synthesis, secretion, degradation and absorption of enamel matrix protein and the transportation of minerals.^1^, ^2^, ^3^ Any abnormal step in this process can lead to defects in the development of enamel.

Transcription factors affect enamel development by regulating the expression of enamel matrix proteins or enzymes.^4^, ^5^, ^6^ Some studies have shown that mutations in transcription factors can also cause defects in the development of enamel.^4^, ^5^, ^6^

Muscle segment homeobox (*Msx*) genes‐encoded proteins perform the function of transcription factors which are expressed at sites of epithelial‐mesenchymal interaction during embryogenesis and control morphogenesis, such as the tooth development.^7^ *Msx1*, *Msx2* and *Msx3* are homologues of *Msx* genes in mammals.^8^ Among them, MSX1 and MSX2 play a major role during the development of the tooth and the craniofacial skeleton, playing an important role in terminal cell differentiation and contributing to the initial patterning of dentition.^9^, ^10^ These studies suggest that MSXs play a pivotal role during organogenesis.

Previous studies have reported that MSX2 may act on dentinogenesis by controlling the formation of mineralized tissues.^11^ The mutation of the human *Msx2* gene causes craniosynostosis syndrome.^12^, ^13^ The absence of *Msx2* leads to compound periodontal osteopetrosis, dentinogenesis imperfecta and amelogenesis imperfecta.^4^, ^14^ MSX2 not only regulates the expression of the enamel protein in ameloblast, but also participates in the regulation of the differentiation and the maintenance of function in the dental epithelium.^15^, ^16^ However, the developmental basis for amelogenesis imperfecta caused by *Msx2*‐mutant remains unclear, including its role in the functional maintenance of the epithelial cells in the enamel organ, enamel defects and the fate of ameloblasts.

To address these questions, we investigated the detailed histological phenotype and underlying mechanism in order to elucidate the mechanism of MSX2 in regulating enamel development.

## 2 MATERIALS AND METHODS

### 2.1 Animal preparation

All animal experiments were conducted according to the Principles of Laboratory Animal Care established by the Ethical Committee of the Institute of Zoology, Chinese Academy of Sciences (Beijing, China). *Msx2* ^+/−^ mice (generated from C57BL/6 mice, GeneID: 17702, Strain official name:C57BL/6‐Msx2^tm1cyagen^) were purchased from Cyagen Biosciences (Guangdong, China) and fed in a pathogen‐free (SPF) condition (12 hours light‐dark cycle; 22 ± 1˚C; 60% humidity). Eight‐week‐old *Msx2* ^+/−^ mice were mated, and the progeny mice genotype was identified by PCR. DNA was extracted from mouse tail tissue and subjected to PCR in which the primers applied were designed to show differences among wild type (*Msx2* ^+/+^, WT, 572 bp), heterozygote (*Msx2* ^+/−^, HET, 572 bp/639 bp) and knockout (*Msx2* ^−/−^, KO,639 bp) mice (Table 1).

**TABLE 1:** Primer sequences and conditions for PCR

Purpose	Name	Primer sequence (5' to 3')	Sizes (bp)	Tm(°C)
Genotype identification	PCR Primers 1	F1:GATAAGACAACTAAGGACCATGC R1:GACCTGACATGGAGTTTCCTATT	639	60
	PCR Primers 2	F2:GGTGAGAGGCACTAAGACCTG R1:GACCTGACATGGAGTTTCCTATT	572	60
qPCR	Msx2	F:CTAAAGGCGGTGACTTGTTTTCG R:CGGCTTCTTGTCGGACATGAG	161	60
	GAPDH	F:TGTGTCCGTCGTGGATCTGA R:TTGCTGTTGAAGTCGCAGGAG	150	60

### 2.2 Histology and immunohistochemistry (IHC)

The dissected mandibles from WT and KO mice and paraffin samples were prepared as previously described^4^ for haematoxylin‐eosin (HE) staining or IHC. For IHC analysis, the sections were treated overnight with primary antibody at 4˚C. Rabbit polyclonal antibody against MSX2, cleavage Caspase‐1 rabbit polyclonal antibody, IL‐1β polyclonal antibody and CK5/6 polyclonal antibody, all of the above antibodies were purchased from Abcam (Abcam, Cambridge, MA, USA). IHC results were detected with the peroxidase substrate kit and the Vectastain ABC Elite kit (Vector Laboratories, Inc, Burlingame, CA, USA) according to the manufacturer's instructions, and haematoxylin stained the nuclei for 4 minutes at room temperature. The experiments were repeated for three times.

### 2.3 Quantitative real‐time PCR (qPCR)

Total RNA was extracted with TRIzol (Takara Biotechnology Co., Ltd., Shiga, Japan). The cDNA was reversely transcribed from total RNA using the MMLV‐RT system (Promega Corporation, Madison, USA), and quantitative real‐time PCR was performed using the SYBR® PrimeScript® kit (Takara Biotechnology) Relative mRNA expression levels were normalized to GAPDH. The primer sequences for real‐time PCR are listed in Table 1. Relative gene expressions were calculated with the 2^−ΔΔCt^ method. Each experiment was performed in triplicate. All the above kits were performed in accordance with the manufacturer's protocol.

### 2.4 Scanning electron microscopy (SEM)

Hemi‐mandibles isolated from WT and KO mice (n = 3 per genotype) were fixed in 10% paraformaldehyde at 4℃ for 20 h. Alcohol gradient dehydration was operated and dried with the critical point drier (Quorum K850). In addition, the samples were embedded in epoxy resin for analysis 25s and air‐dried at room temperature. The specimen was coated by palladium gold and observed under a scanning electron microscope (ZEISS EVO LS 15; Carl Zeiss AG, Jena, Germany) at 10 kV.

### 2.5 Micro‐computed tomography (µCT) analysis

The mandibles from WT and KO mice were isolated, and the soft tissue (around the bone and teeth) was removed. Specimens were fixed in 10% formaldehyde (pH 7.5) solution at 4℃ for 20 h and washed at 4℃ overnight. The mandibles were scanned using the imaging system micro‐CT 50 (Scanco medical, Bassersdorf, Switzerland) with a thickness of 9 μm under a scanning parameter of 70kV and 114 mA. After the scanning, VGStudio Max Version 2.0 software was used to analyse the scanning data, and then, the whole structure and different sections of mandible were analysed and evaluated.

### 2.6 Detection of pyroptosis in mouse mandibles by TUNEL

TUNEL (terminal deoxynucleotidyl transferase dUTP nick end labelling) assays were performed in mandibles from WT and KO mice with the TUNEL Bright Green Apoptosis Detection Kit (Vazyme Biotech Co., Ltd, Nanjing, China) according to the manufacturer's instructions. Sections for TUNEL staining were prepared using the same treatment method as tissue sectioned for HE staining. The sections were washed in PBS and counter stained with DAPI. Finally, the sections were dehydrated and mounted.

### 2.7 Western blotting (WB) analyses and ELISA

The mandibles from WT and KO mice were separated and ground in liquid nitrogen and then lysed with cell lysis buffer (Beyotime Biotechnology), and lysates were centrifuged at 10 000 *g* for 5 minutes at 4˚C. Total protein from the supernatant was quantified using an enhanced BCA protein assay kit (Beyotime Biotechnology). Western blotting analyses of protein expression were also performed as previously described.^4^ The primary antibodies were rabbit anti‐cleavage Caspase‐1 monoclonal antibody (Abcam), and β‐actin antibody (Santa Cruz Technology, Dallas, TX, USA) was used as an internal control. The Amersham enhanced chemiluminescence kit (GE Healthcare Life Sciences, Little Chalfont, UK) was used for detection. The Western blotting analyses were performed in triplicate.

ELISA, 5 ng total proteins were used for ELISA to detect cleaved IL‐1β activity (Beyotime Biotechnology) according to the manufacturer's protocol.

### 2.8 Statistical analysis

The data were analysed and compared using two‐way ANOVA analysis and represented as the mean ± SD. Student's t test was used for the significant differences between two groups. Dunnett's test evaluated multiple comparisons when the F test result was significant. A value of P < 0.05  was considered to indicate a statistically significant difference. Asterisks were considered to indicate statistically significant differences (*P < 0.05; **P < 0.01; ***P < 0.001) from the control groups.

## 3 RESULTS

### 3.1 Validation of MSX2 ablation in teeth of KO mice

**FIGURE 1:**
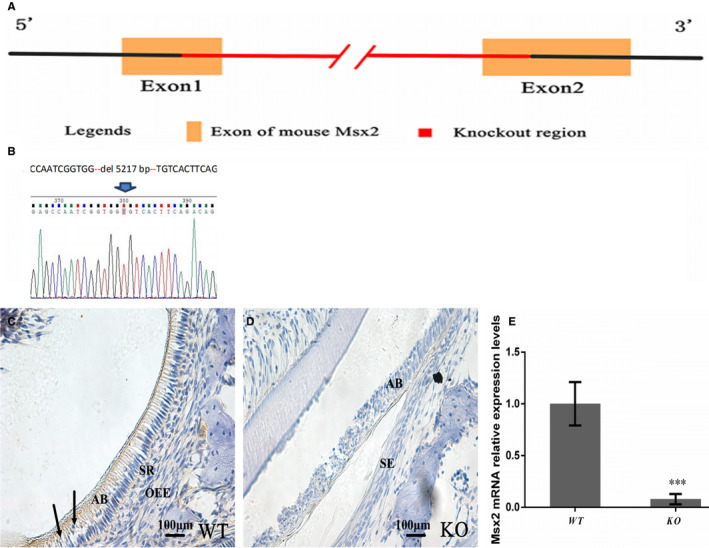
Construction and validation of the Msx2^‐/‐^ (KO) mouse model. A, The 5217 bps from exon 1 and exon 2 were deleted in the Msx2 gene. B, The deletion of the Msx2 gene was confirmed by sequencing. The entire CDS area for Msx2 has been deleted. Validation of Msx2 inactivation in P10 (postnatal day 10) mice by IHC. D, There was no MSX2 detected in the molar enamel organ of KO mice, (C) while ameloblasts and the enamel epithelium (SI, SR and OEE) showed positive staining. E, The Msx2 expression at the mRNA level was significantly decreased in the mandible of KO mice and which was determined at P10 by RT‐qPCR. ***P < 0.001. n = 3. AB, ameloblast; OEE, outer enamel epithelium; SE, squamous epithelium; SR, stellate reticulum. Scale bars = 100 μm

*Msx2* ^+/‐^ mice were generated by Cyagen Biosciences. Figure 1A illustrates the details of the gene targeting and genotyping strategy. The region from exon 1 and exon 2 was deleted in the *Msx2* gene. Furthermore, 5217 bp of *Msx2* gene were successfully deleted as determined by sequencing (Figure 1B). Therefore, ablating 5217 bp in the *Msx2* gene should result in functional loss of the MSX2 protein. Offspring mice from *Msx2* ^+/−^ × *Msx2* ^+/−^ crosses had a normal Mendelian distribution of genotypes (25% *Msx2* ^+/+^, 50% *Msx2* ^+/−^and 25% *Msx2* ^−/−^).

The deletion of *Msx2* expression at RNA level and protein level was detected by qPCR and IHC staining, respectively. WT mice showed positive staining for MSX2 in ameloblasts and the enamel epithelium which surround the ameloblasts, including the outer enamel epithelium (OEE), the stellate reticulum （SR）and stratum intermedium (SI). Especially in ameloblasts of a mature stage, the expression signal is stronger (arrows, Figure 1C). However, no positive staining for MSX2 was observed in ameloblasts and the enamel epithelium in KO littermates (Figure 1D). Moreover, we further confirmed that the *Msx2* expression at the mRNA level was significantly decreased in the mandible of KO mice (Figure 1E). These results indicated that the *Msx2* KO mouse model was successfully established.

### 3.2 Loss of *Msx2* results in enaa‐mel defects

**FIGURE 2:**
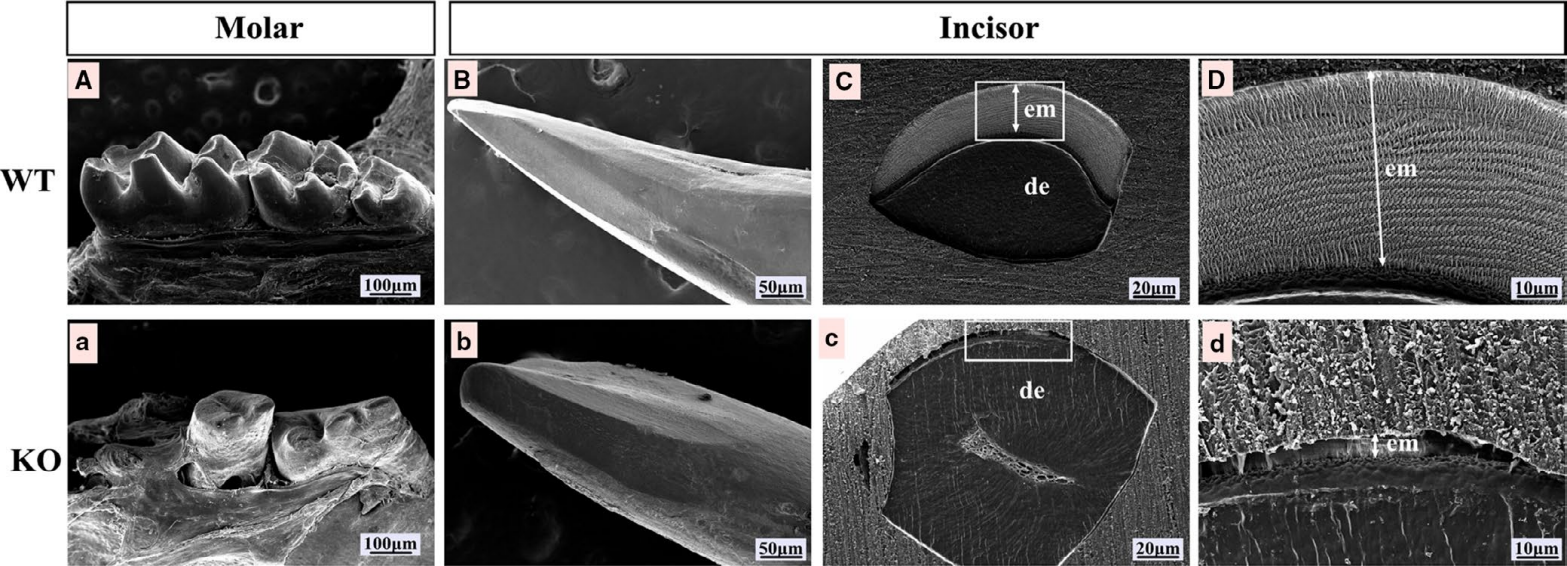
KO mice teeth suffered severe abrasion and defect of enamel. Scanning electron microscopy revealed that severe abrasion appeared on molars and incisors from 4‐week‐old KO mice (a, b) compared to the effects observed in the WT mice (A, B). Sections from incisors show that severe defects appeared in KO mice (c, d) compared with WT mice (C, D). The KO mice presented with only a thin mineral layer without enamel rods. em‐enamel, de‐dentin. Scale bars: 100 μm (A, a), 50 μm (B, b), 20 μm (C, c), 10 μm (D, d)

We found that there was no obvious difference between WT and KO mice in the phenotype of the molar from 15‐day‐old mice, while the molars of 4‐week‐old KO mice were severely worn comparing with that of WT mice. We further examined the enamel structure of incisors and molars by SEM (Figure 2). Compared with WT mice (Figure 2A–D), the molars and incisors of KO mice had rough surfaces, severe wear and even missing tooth tips (Figure 2 a,b). Through observation of the transverse section of incisors, it was found that KO mice had missing enamel rods, and only a thin layer of rod‐less enamel covered the dentin surface (Figure2 c,d).

**FIGURE 3:**
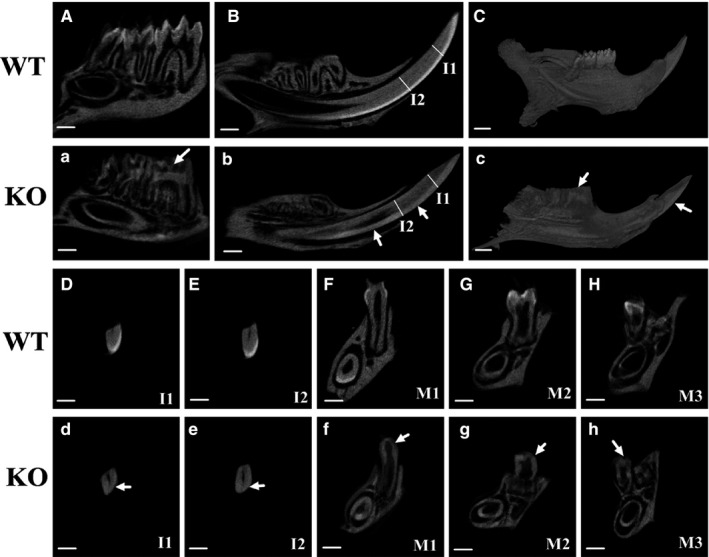
No mature enamel was formed during development in KO mice. Micro–computed tomography analysed hemi‐mandibles from 4‐week‐old mice. 9‐μm sagittal sections (A, B, a, b) and transverse sections (D‐H, d‐h) through the mandibular incisors (D, E, d, e) and molars (F‐H, f‐h) were analysed. The enamel of incisors and molars in WT mice showed a sharp contrast with dentin, while the incisors and molars in KO mice displayed defect of enamel (arrows). Three‐dimensional reconstruction of the mandibles (C, c). I1: incisor transverse section 1, I2: incisor transverse section 2, M1: the first molar, M2: the secondary molar, M3: the third molar

To further clarify the enamel hypomaturation defects of KO mice, the hemi‐mandibles from 4‐week‐old mice were scanned by micro‐computed tomography. Sagittal sections and transverse sections through the mandibular incisors (Figure 3B,D,E) and mandibular molars (Figure 3A,F,G,H) showed strong contrast between enamel and dentin in WT mice (Figure 3A‐H). However, in KO mice (Figure 3a‐h) the mature enamel layer was not even observed in the incisor (Figure 3b,d,e) or the molars (Figure 3a,f,g,h). In KO mice, three‐dimensional reconstruction showed that the enamel exhibited a low mineral density, and molars have been almost ground down (Figure 3c) compared to that of WT mice (Figure 3C). These findings demonstrated that MSX2 plays an important role in enamel mineralization.

### 3.3 Ameloblast pyroptosis resulted in the inability of enamel to mineralize during the transition stage in KO mice

**FIGURE 4:**
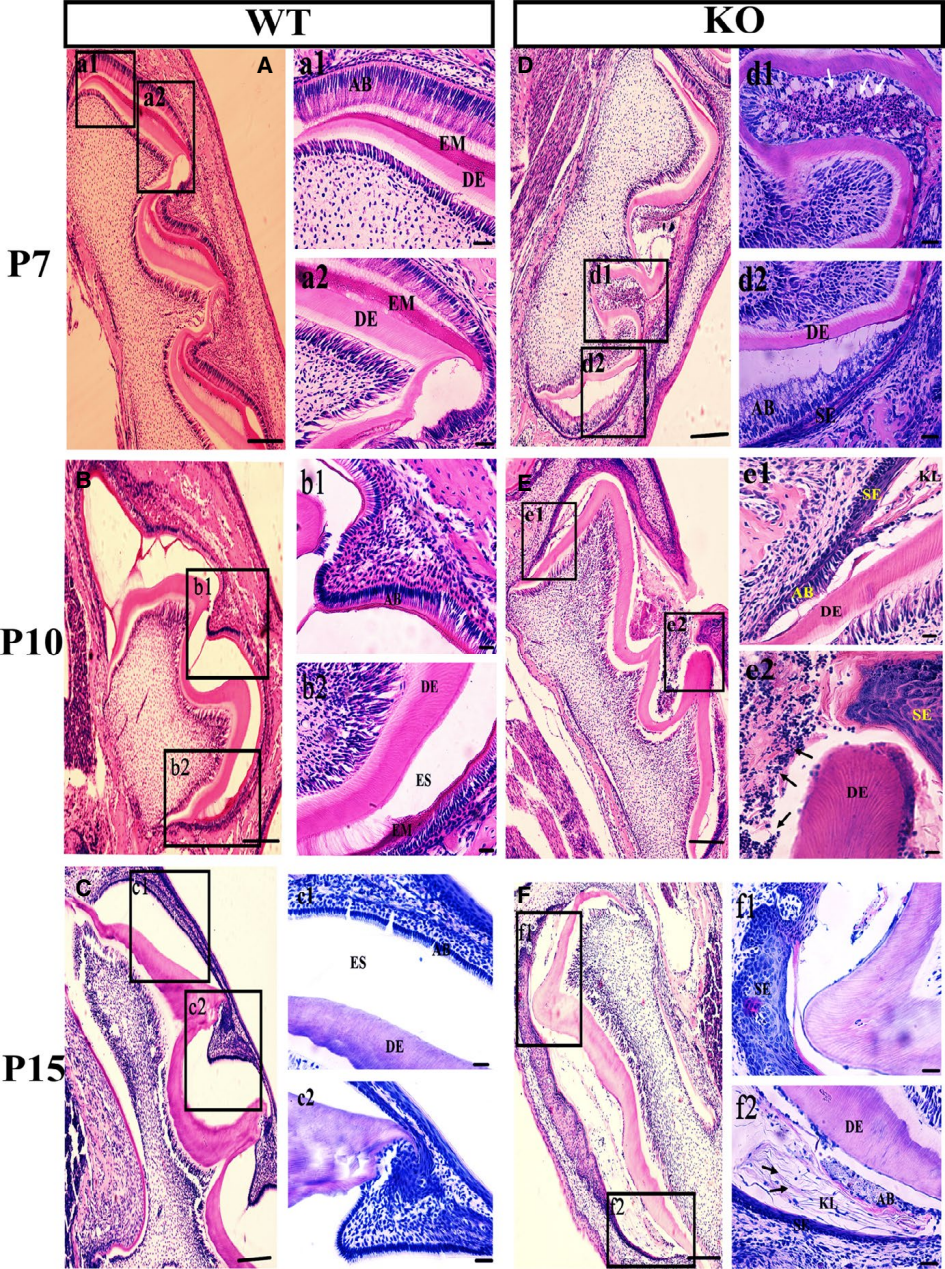
Histological analysis of the mandibular molars. Histological analysis was performed on the first molars of KO mice (D, E, F) and WT mice (A, B, C) at P7, P10 and P15. WT mice were used as control, and significant differences were found in the enamel organ of KO mice. At P7, KO mice showed normal ameloblast morphology only at the early enamel secretion stage, with columnar shape cells secreting a small amount of enamel (D, d2). However, in the middle stage of enamel secretion, squamous epithelial (SE) proliferation occurred in the enamel epithelium (OEE), the stellate reticulum (SR) and stratum intermedium (SI) of KO mice (D, d2), while ameloblast morphology was abnormal, and inflammatory cell aggregation occurred (arrows, D, d1). At P10, squamous cell proliferation was more pronounced with partial keratinization (arrows, E, e1), and mass ameloblast death with more inflammatory cells gathering in the enamel organ of KO mice (arrows, E, e2). At P15, Ameloblasts disappeared while a few inflammatory cells remained (arrows, F, f2). There were lamellar squamous cells in the enamel organ of KO mice (F, f1). AB, ameloblast; EM, enamel; DE, dentin; ES, enamel space; KL, keratin layer; SE, squamous epithelium

**FIGURE 5:**
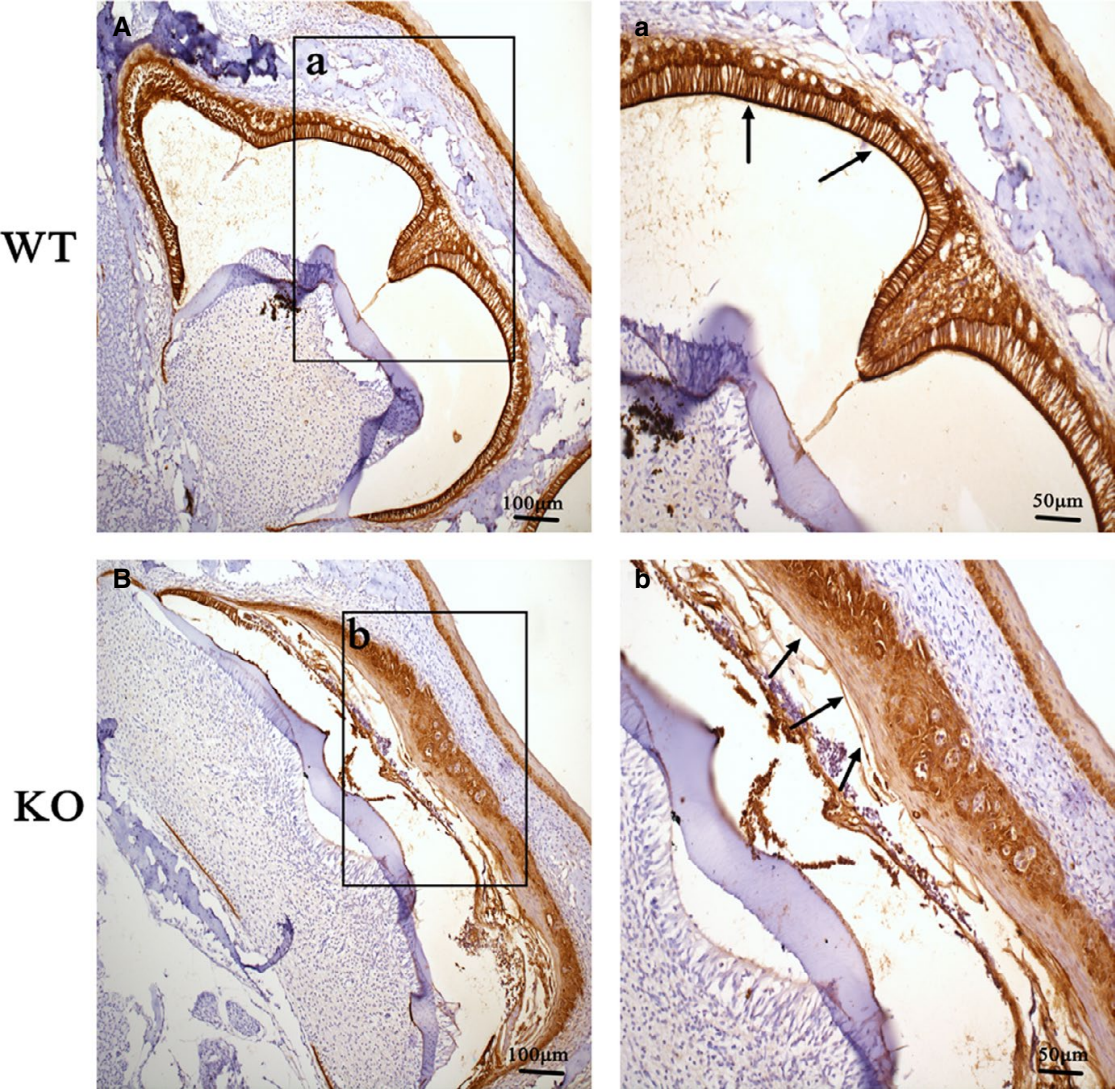
Squamous epithelial proliferation of the enamel epithelium in the enamel organ of KO mice.CK5/6 expression analysis was performed on the first molars of WT mice (A) and KO mice (B) at P10 by immunohistochemistry. WT mice were used as control, and squamous hyperplasia proliferation of the enamel epithelium was significant, and the keratinization phenomenon in squamous epithelial cells appeared near ameloblast in KO mice (arrows, b)

To further clarify the cause of enamel defects in KO mice, HE staining was used to observe the histological changes during enamel formation (Figure 4). The experimental results in KO mice showed that a small amount of enamel was secreted in the early stage of enamel secretion, but no mineralization occurred in the mature stage, which led to severe enamel defects (Figure 4D). Compared with WT mice, squamous epithelial hyperplasia of the enamel epithelium has appeared in the epithelial cell layer formed by OEE, SR and SI of KO mice at P7 (Figure 4D). At the same time, ameloblasts also changed from polarization to depolarization, and enamel secretion stopped (Figure 4D). CK5/6, a marker of squamous epithelial cells, was used to confirm that the enamel epithelium had squamous epithelial proliferation in the enamel organ of KO mice by immunohistochemistry (Figure 5B). Squamous hyperplasia proliferation of the enamel epithelium was serious, and the keratinization phenomenon in squamous epithelial cells appeared near ameloblast in KO mice (Figures 4E and 5B). At P15, keratinization of squamous epithelial cells further intensified (Figure 4F).

**FIGURE 6:**
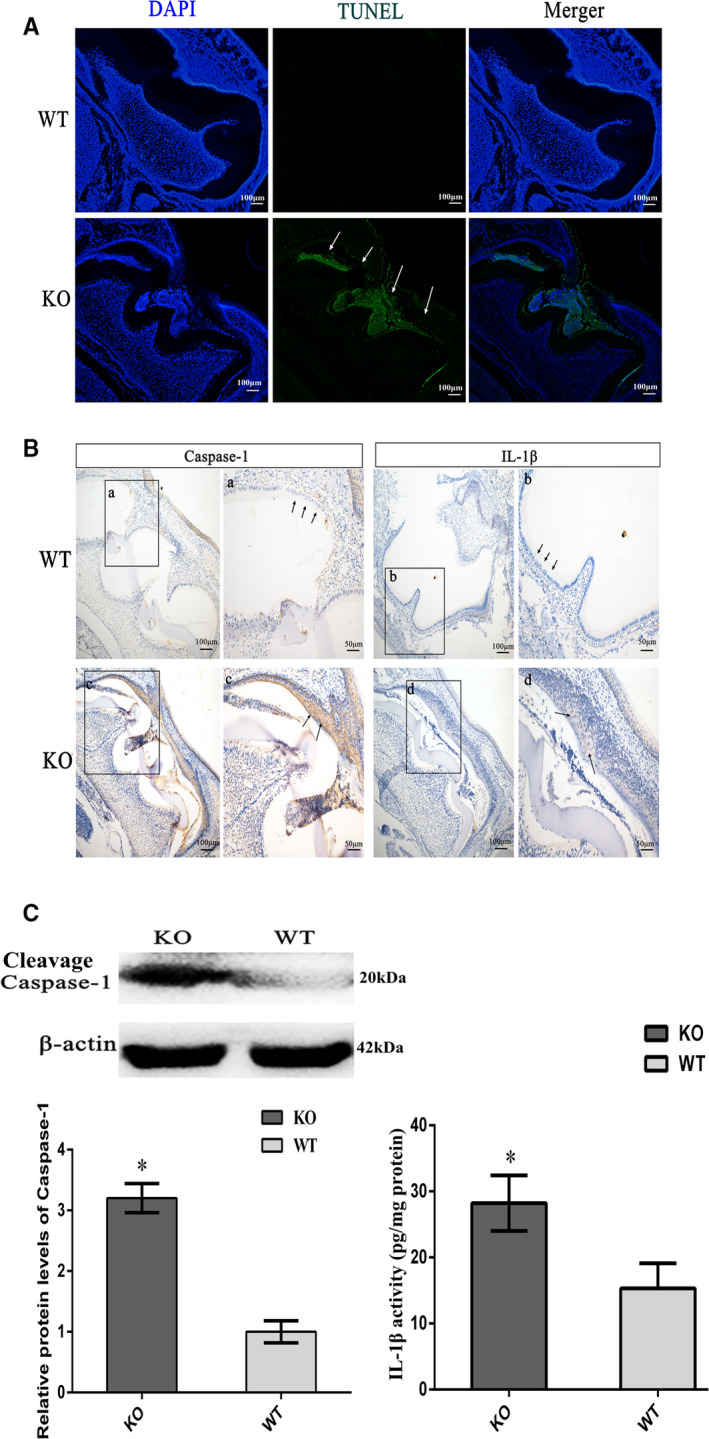
Absence of Msx2 induced the pyroptosis of ameloblasts. A, At P10, the pyroptosis of ameloblast in KO mice was shown with TUNEL staining in green (arrows), while no obvious staining was detected in ameloblasts of WT mice. The nuclei were stained with DAPI in blue. B, As a marker of pyroptosis, both cleavage Caspase‐1 and cleaved IL‐1β expression were significantly increased in the enamel organ of KO mice compared with that of WT mice by immunohistochemistry (arrows, B, c, d). Cleavage Caspase‐1 expression was determined by WB (C), and cleaved IL‐1β activity was performed for the quantification by ELISA (D). *P < ​ 0.05. n = 3

In addition, the HE staining results indicated that ameloblasts and epithelium adjacent to ameloblasts in the enamel organ were dead at the mature stage, and a large number of inflammatory cells had accumulated (Figure 4D‐F). At P10, a large number of ameloblast**s** underwent pyroptosis in KO mice, which was further confirmed by TUNEL staining (Figure 6A). In the process of pyroptosis, the activation of cleavage Caspase‐1 further promotes the maturation of IL‐1β. IL‐1β is released to the outside of the cell, and then, IL‐1β collects inflammatory cells and triggers inflammatory response. Both cleavage Caspase‐1 and cleaved IL‐1β were higher in the enamel organ of KO mice than that of WT (Figure 6B), as determined by WB (Figure 6C) and ELISA (Figure 6D) separately, indicating that pyroptosis occurred in the enamel organ of KO mice.

The above results showed that the loss of *Msx2* could induce the keratinization of a large number of epithelial cells near ameloblasts, which caused ameloblasts to separate from the epithelial layers. We speculate that the pyroptosis of ameloblasts may be due to the obstruction of nutrient access. It is suggested that the loss of *Msx2* could induce the proliferation and keratinization of squamous epithelial cells and cause pyroptosis of ameloblasts and the aggregation of inflammatory cells. Therefore, MSX2 is a necessary factor to maintain the state and function of epithelial cells.

## 4 DISCUSSION

MSX2, as a transcriptional repressor, may establish a balance between the survival and apoptosis of neural crest‐derived cells which are required for proper craniofacial morphogenesis.^13^, ^17^ MSX2 may also play a role in inhibiting cell proliferation and promoting apoptosis.^18^, ^19^ Under physiological and pathological conditions, MSX2 plays an important role in space‐time limitation of enamel protein production and regulation of Enamel Quality (thickness and mineralization level).^20^

MSX2 functional deletion mutation in a mouse model caused impaired enamel formation.^21^ Alveolar bone necrosis is induced by experimental periodontitis in *Msx2* mutant mice.^22^ It can be seen that MSX2 plays an important role in the development of enamel and alveolar bone. Although it has been reported that MSX2 can prevent stratified squamous epithelium formation in the enamel organ,^16^ it is poorly understood that the underlying mechanism by which MSX2 regulates amelogenesis. Therefore, the detailed histological and molecular analyses were performed in *Msx2* null mice. We have found that the loss of *Msx2* results in serious defects in enamel mineralization by SEM and micro‐computed tomography. It was found that there is no enamel rod in the enamel area of *Msx2* ^‐/‐^ mice, and only a thin mineralized layer prone to wear. Our results are consistent with those of previous studies.^4^ In the epithelial life cycle, the levels of *Msx2* expression are negatively correlated with the level of enamel protein secretion.^23^ *Msx2* ^‐/‐^ mice display compound phenotype characteristics of enamel defects.^4^, ^16^, ^21^

To further clarify the cause of enamel loss in *Msx2* ^‐/‐^ mice, the histology of enamel formation stage was observed through HE staining. The results showed that *Msx2* ^‐/‐^ mice only had a small amount of secreted enamel matrix protein in the early stage of enamel formation, but no mineralization occurred, resulting in severe enamel defects.

There are many reasons for enamel defects in *Msx2* ^‐/‐^ mice. For example, the loss of *Msx2* may cause the abnormal secretion of enamel matrix protein, which then affects the formation of enamel. This is consistent with the known repressive transcriptional activity of MSX2 on enamel matrix protein genes such as enamelin, amelogenin and ameloblastin in enamel.^4^, ^14^ We found that although a small amount of enamel matrix protein was secreted in the early stage, no mineralized enamel was eventually formed, which indicated that the key points of enamel defects caused by the loss of *Msx2* are the mineralization deficiency of enamel at the mature stage. On the other hand, in the *Msx2* ^‐/‐^ mice, it was also found that the epithelial cell layer adjacent to ameloblasts had advanced squamous epithelialization in the later stage of enamel formation, which was consistent with previous research results.^16^

We found that the epithelial cells nearing ameloblasts appeared to be hyperplasic squamous cells at postnatal day 15 in WT mice; however, these cells became hyperplasic squamous cells at postnatal day 7 in *Msx2* ^‐/‐^ mice. In addition, the squamous epithelial cells had severe keratinization at postnatal day 10 in *Msx2* ^‐/‐^ mice. Keratinization resulted in the separation of ameloblasts from the epithelial cell layer, which led to a high degree of cell death, presumably due to a lack of nutrition, and then a large amount of inflammatory cell aggregation, which defines the pyroptosis phenomenon. At this time, HE staining results also showed that the ameloblasts displayed homogeneous red staining of unstructured material, and nuclear staining disappeared. Both cleavage Caspase‐1 and IL‐1β were higher in the enamel organ of *Msx2* ^‐/‐^ mice than that of wild type. These observations are characteristic of pyroptosis.^24^, ^25^, ^26^, ^27^

Our data delineate a putative mechanism: the absence of *Msx2* causes the proliferation and keratinization of squamous epithelial cells, which caused ameloblasts to separate from the epithelial layers and pyroptosis occurred in the enamel organ from the early stage of enamel secretion. Our results enrich the knowledge of the function of MSX2 during enamel development. Nevertheless, in order to explore the specific regulatory mechanism of MSX2 in enamel development, it is necessary to carry out further research, including how MSX2 induces squamous cell hyperplasia of the enamel epithelium, and what is the regulatory mechanism of MSX2 in mature ameloblast cells.

## CONFLICT OF INTEREST

The authors confirm that there are no conflicts of interest relevant to this article.

## AUTHOR CONTRIBUTIONS

**Zhang Juanjuan:** Methodology (lead). **Xu Ying:** Methodology (equal). **Zhao Ying:** Formal analysis (supporting). **Bai Jingkun:** Funding acquisition (equal). **Xu Mengge:** Software (equal). **Li Chuanji:** Resources (equal). **Li Jinyue:** Resources (equal). **Ren Yong:** Formal analysis (equal). **Xu Chang:** Data curation (equal). **Gao Yuguang:** Data curation (equal). **Sun Yan:** Writing‐review & editing (equal). **Liu Xiaoying:** Writing‐original draft (lead).

## Data Availability

Data sets used and analysed during the current study are available from the corresponding author on reasonable request.
